# The Inhibition of the Degrading Enzyme Fatty Acid Amide Hydrolase Alters the Activity of the Cone System in the Vervet Monkey Retina

**DOI:** 10.3390/brainsci11111418

**Published:** 2021-10-27

**Authors:** Joseph Bouskila, Maxime Bleau, Catarina Micaelo-Fernandes, Jean-François Bouchard, Maurice Ptito

**Affiliations:** 1 School of Optometry, University of Montreal, Montreal, QC H3T 1P1, Canada; maxime.bleau.1@umontreal.ca (M.B.); catarina.fernandes@umontreal.ca (C.M.-F.); jean-francois.bouchard@umontreal.ca (J.-F.B.); maurice.ptito@umontreal.ca (M.P.); 2 Behavioral Science Foundations, Estridge KN0101, St-Kitts and Nevis; 3 Department of Neuroscience, Copenhagen University, 2200 Copenhagen, Denmark; 4 Department of Neurology and Neurosurgery, Montreal Neurological Institute, McGill University, Montreal, QC H3A 2B4, Canada

**Keywords:** cone pathway, flicker electroretinogram, URB597, endocannabinoids, FAAH, retina, vervet monkeys

## Abstract

Recent studies using full-field electroretinography (ffERG) that triggers a non-specific mass response generated by several retinal sources have attributed an important role for cannabinoid receptors in mediating vision in primates. Specific cone-mediated responses evoked through the photopic flicker ERG appear to be a better way to validate the assumption that endogenous cannabinoids modulate the cone pathway, since FAAH is mainly expressed in the vervet monkey cone photoreceptors. The aim of this study is two-fold: (1) to use the photopic flicker ERG to target the cone pathway specifically, and (2) use URB597 as a selective inhibitor of the endocannabinoid degrading enzyme Fatty Acid Amide Hydrolase (FAAH) to enhance the levels of fatty acid amides, particularly anandamide. We recorded ERGs under four different flicker frequencies (15, 20, 25, and 30 Hz) in light-adapted conditions after intravitreal injections of URB597. Our results show that intravitreal injections of URB597, compared to the vehicle DMSO, increased significantly ffERG amplitudes at 30 Hz, a frequency that solely recruits cone activity. However, at 15 Hz, a frequency that activates both rods and cones, no significant difference was found in the ERG response amplitude. Additionally, we found no differences in implicit times after URB597 injections compared to DMSO vehicle. These results support the role of molecules degraded by FAAH in cone-mediated vision in non-human primates.

## 1. Introduction

Cannabinoid receptors are localized throughout the retina of several animal species [1,2], including primates [3,4,5,6,7]. In monkeys, the cannabinoid receptor type 1 (CB1R) is mainly expressed in the cones of the central retina [8], whereas the cannabinoid receptor type 2 (CB2R) is localized in the retinal glia (Müller cells) [9]. As for the putative cannabinoid receptor G-protein coupled receptor 55 (GPR55), it is exclusively expressed in rod photoreceptors [10]. The degrading fatty acid amide hydrolase (FAAH) and synthesizing (NAPE-PLD) enzymes related to CB1R are also expressed in the cone system of several animal species [8,11,12]. Recently, TRPV1 that responds to some cannabinoid agents has been shown to be highly expressed in the horizontal pathway of the vervet monkey retina, namely the horizontal and amacrine cells [13].

Growing evidence indicates a fundamental role for the endocannabinoid (eCB) system in regulating primate retinal function [14]. Full-field electroretinogram (ffERG) recordings represent a useful clinical tool to evaluate the integrity of the retina. The ffERG is a non-specific mass response made out of several retinal sources that are summed throughout the retina, and this method has been used for studying the eCB system in several animal species including mice and primates [1,3,4,14,15,16,17]. However, less is known about eCB signaling in cone responses using the photopic flicker ERG, especially in the 30 Hz flicker frequency that is known to activate mainly the cone system [18]. Manipulating the eCB system by blocking the activity of CB1R using intravitreal injections of the specific antagonist AM251 alters the global ffERG in the vervet monkey [14]. These results led to the suggestion that, in the primate model, CB1R acts as a mediator of central retinal function [4].

There are two important parameters generally used to analyze the flicker ERG waveforms, the amplitude and the implicit time (latency) [19,20,21,22]. Retinal diseases that target mainly the cone system typically display reduced amplitude responses and altered implicit times [19,23]. Pharmacological studies using the photopic flicker ERG to isolate receptor and post-receptor responses have shown that this test is generated dominantly by post-receptor components and have provided a better understanding of how these elements, particularly the ON and OFF bipolar cells, interact to shape the steady state flicker ERG response [24,25].

Molecules that can change levels of eCBs are interesting pharmacological targets for treatments of retinal diseases. Imbalances in the retinal eCB system levels may cause or exacerbate pathological disorders such as glaucoma, macular degeneration, diabetic retinopathy, and retinitis pigmentosa [26,27,28,29]. The endogenous ligands of CB1R (anandamide or AEA; [30], and 2-arachidonoylglycerol or 2-AG; [31,32]) are produced “on demand” and act retrogradely by binding to cannabinoid receptors [33]. The eCBs, among other bioactive lipids such as some simple esters and amides with long unsaturated acyl chains, are majorly degraded by the fatty acid amide hydrolase (FAAH) enzyme [34,35], and as such, inhibition of FAAH leads to an accumulation of some eCBs. Significantly, high levels of AEA were found in the retina of diabetic retinopathy patients [28], suggesting a dysfunction of the degrading enzyme FAAH. The molecule URB597, a potent, irreversible, and selective inhibitor of FAAH that has been extensively studied [36,37,38,39,40,41,42], can mimic the high levels of eCBs in the retina.

In general, most of the studies have used either full-field or pattern ERGs that measure the global retinal response by activating both cone and rod photoreceptors [43,44,45,46]. In this study, we specifically investigated the contribution of the enzyme FAAH on solely the cone system by using photopic flicker ERG recordings. We recorded, in the vervet monkey, light-adapted flicker ERGs before and after intravitreal injections of the FAAH inhibitor URB597. Since: (1) the inhibition of FAAH by URB597 leads to increase eCB levels and thus activation of CB1R, (2) the expression of CB1R is mainly expressed in the cone system [8], and (3) the decrease neurotransmitter release from cones mimics light activation, we hypothesized that URB597 will increase the cone-mediated photopic flicker ERG response by enhancing the activity of CB1R in cones. We found that indeed, interfering with the endogenous levels of bioactive lipids, particularly the eCBs such as AEA, with URB597 increases the amplitude of the photopic flicker ERG, but not the latency. These results support the role of eCBs in cone-mediated vision in monkeys [8,14].

## 2. Materials and Methods

**Animals.** A total of 13 vervet monkeys (*Chlorocebus sabaeus*) were used for this study: six monkeys received a monocular intravitreal injection of dimethyl sulfoxide (DMSO) solution, the vehicle) and seven monkeys of URB597 (Table 1). The animals were born and raised in enriched environments in the laboratories of the Behavioral Science Foundation (St-Kitts, West Indies). The animals were fed with primate chow (Harlan Teklad High Protein Monkey Diet; Harlan Teklad, Madison, WI, USA) and fresh local fruits, with water available ad libitum.

**Animal preparation.** The method for ERG recordings in the vervet monkey has been previously described [14,16,47] and is briefly reported here. The animals were sedated with an intramuscular injection of a mixture of ketamine (10 mg/kg; Troy Laboratories, Glendenning, New South Wales, Australia) and xylazine (1 mg/kg; Lloyd Laboratories, Shenandoah, IA, USA). In this condition, the pupils were fully dilated to approximately 9 mm in diameter and the accommodation reflex was paralyzed with topical application of 1% tropicamide (Mydriacyl) and 2.5% phenylephrine hydrochloride (Mydfrin) (Alcon Laboratories, Fort Worth, TX, USA). The eyes were treated with 0.5% proparacaine hydrochloride (Alcaine; Alcon Laboratories, Fort Worth, TX, USA) to anesthetize the cornea and then protected by application of 2.5% methylcellulose (Gonak; Akorn, Inc., Buffalo Grove, IL, USA) to prevent corneal drying. Recording sessions lasted approximately 1 h for each animal, after which they received a tobramycin antibiotic ointment (Sandoz Laboratories, Boucherville, QC, Canada), and 3 days after recovery, they were returned to their prior naturalistic setting.

**Figure 1:**
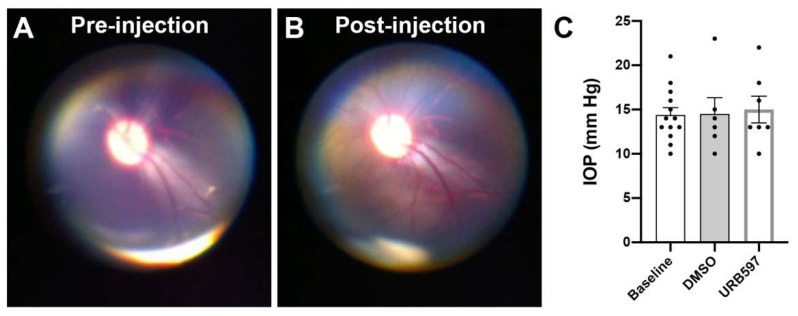
Fundus observation and assessment of intraocular pressure (IOP) with individual values. No differences in the fundus of the eyes before (**A**) or after a typical intravitreal injection (**B**). IOP values did not differ at baseline (before intravitreal injections), post-DMSO injection or post-URB597 injections (**C**). Error bars represent standard error of the means (SEM).

**Administration of drugs.** The intravitreal injections were performed in the right eye of all monkeys under sedation, the fellow eye serving as a non-injected control. After inspection and examination of the eyes and lids, a topical anesthetic was applied over the injection site. The conjunctival and corneal surfaces were then moistened with methylcellulose (Moisture Eyes, Bausch Lomb, Rochester, NY, USA) for 3 min. The eye was covered with sterile coatings and a Barraquer eye speculum (1.75 in., 10 mm wide small blades; Storz Ophthalmics, St. Louis, MO, USA) held the eyes open. The URB597 was purchased from Cayman Chemicals (item number 10046, Ann Arbor, MI, USA). With a 30 G needle, 50 μL of URB597 solution (1 mg/mL; for a final concentration of 0.01 µg/µL) was injected into the vitreous cavity, 2 mm posterior to the corneal limbus. A similar volume (50 μL) of the vehicle DMSO solution (final concentration 1.5% *v*/*v*) was injected into the vitreous of the right eye in six monkeys in order to rule out the possibilities that the vehicle, the injection per se and/or changes in intraocular pressure caused the effects attributed to the drug (see Figure 1). When the needle was removed, the injection site was compressed for a minute using a sterile cotton swab to avoid reflux. For the whole subsequent week, topical tobramycin ointment was administered to the eye that had been injected, twice daily for 4 days.

**Ophthalmoscopy and Intraocular pressure.** Direct ophthalmoscopy, using the panoptic ophthalmoscope (Welch Allyn, Skaneateles Falls, NY, USA) attached to an iPhone camera (iExaminer, Welch Allyn, Skaneateles Falls, NY, USA), was performed by the same experimenter to observe the fundus and vascularization of the eyes before and immediately after the intravitreal injection to ascertain that the retina was not damaged by the injection. Intraocular pressure (IOP) was also monitored before and 2–3 min after the intravitreal injection by applanation tonometry (TonoPen XL; Mentor, Norwell, MA, USA).

**Visual stimulation.** Visual stimulation was produced with an UTAS BigShot Ganzfeld light source (UTAS E-3000 electrophysiology equipment; LKC Technologies, Inc., Gaithersburg, MD, USA) that was placed in front of the animal’s face. ERGs were recorded and averaged from the right eyes before and 5–10 min after injection as detailed below. Using a steady white background-adapting field (30 cd/m^2^) presented inside the Ganzfeld, ERGs were evoked by a flicker LED flash luminance of 2.57 cd.s.m^2^ (0.4 log cd.s.m^2^) delivered in full-field conditions. The flash intensity and background luminance were calibrated using a research radiometer (IL1700 Photometer; International Light Inc., Newburyport, MA, USA) with a SED033 detector placed at 36 cm from the source.

**Flicker ERG recording and analysis.** Light-adapted flicker ERG was used to specifically target cone function at 30 Hz. All experimental protocols followed the general guidelines of the International Society for Clinical Electrophysiology of Vision (ISCEV), specifying the 30 Hz light-adapted response to a rapidly repeated stimulus. ERG recordings and signal processing were recorded with contact lens electrodes lying across the center of the cornea of each eye moistened with 1% carboxymethylcellulose sodium (Refresh Celluvisc, Allergan Inc., Markham, ON, Canada). The corneal contact lens electrode (Jet electrodes; Diagnosys LLC, Lowell, MA, USA) was equipped with four small posts on the convex surface in order to keep the eyelids open. Reference and ground gold disc electrodes (model F-E5GH; Grass Technologies, Astro-Med, Inc., West Warwick, RI, USA) were kept in place with adhesive paste (Ten20 conductive EEG paste; Kappa Medical, Prescott, AZ, USA) at the external canthi and forehead, respectively. Up to five waveforms were averaged to reduce variability and background noise. For every flash frequency, a peak and through response is generated. The amplitude of the flicker ERG was defined as the through-to-peak value in microvolts (µV) of a typical wave, whereas the implicit time (latency) of the photopic flicker response was defined from the midpoint of the stimulus flash to the following peak (avoiding the initial waveform) [18]. The average amplitudes and implicit times of the flicker ERG curves were obtained at each frequency by the EMWin software (LKC Technologies, Gaithersburg, MD, USA). This technique estimates implicit times based on finding the center of the response; a method that has been proven effective in prediction of neovascularization of the iris in central retinal vein occlusion [48]. When single ERG values were not detected, an amplitude or latency value was generated using the regression imputation method. Retinal response diagrams were drawn using Adobe Illustrator and processed in Adobe InDesign (Adobe Systems, software version CC; San Jose, CA, USA).

**Statistical analysis.** Recorded ERG responses pre- and post-injection were analyzed with a 2 (between subject factor; administered drug: DMSO versus URB597) × 2 (within subject factor; time of recording: pre-versus post-injection) × 4 (within subject factor; frequencies: 15, 20, 25 and 30 Hz) mixed factor ANOVA.

## 3. Results

**Ophthalmoscopic observations and intraocular pressure.** No changes in the fundus of the eyes were observed by the same experimenter after each intravitreal injection performed in all animals (Figure 1A,B), indicating that the retina kept its integrity and was not affected by the injection. Immediately following the eye injection, there was a slight increase in IOP that subsided for a few minutes before returning to normal. When the IOP returned to pre-injection level, ERG recordings started. There were no significant pupil size differences (9 mm dilation before and after injections) and IOP (14.4 ± 2.3 mm Hg pre-injection, 14.5 ± 3.0 mm Hg after DMSO injection, and 15.0 ± 3.1 mm Hg after URB597 injection). Figure 1C illustrates the assessment of IOP with individual values.

**Figure 2:**
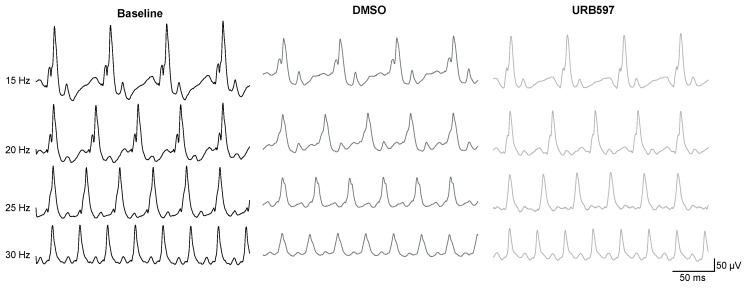
Schematic raw flicker ERG traces obtained in a representative monkey at baseline (**left**), and following vehicle injection (**middle**) or URB597 injection (**right**). Responses were elicited at 4 different flicker frequencies (15 Hz, 20 Hz, 25 Hz, and 30 Hz). Flash intensity was constant at 2.57 cd.s.m^−2^. Note the large difference at 30 Hz between DMSO and URB597. Scale is given in the inserts.

Photopic flicker ERG. The photopic flicker ERG was recorded before, and following intravitreal injection of DMSO or URB597 at 4 different frequencies, ranging from 15 Hz to 30 Hz, obtained in light-adapted conditions (30 cd/m^2^) with standard intensity light flashes (2.57 cd.s/m^2^). Figure 2 shows raw flicker ERG traces for DMSO and URB597 treated eyes.

**Figure 3:**
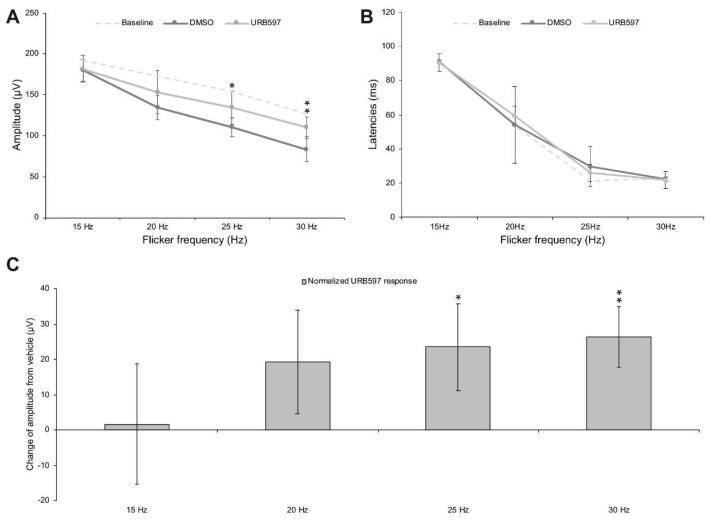
Frequency/response relationship of the flicker ERG following intravitreal administration of URB597. Peaks in amplitude values were observed at 15 Hz and then slowly decreased at 30 Hz. URB597 was significantly higher than DMSO at 30 Hz (**A**). Implicit times decreased with increasing frequency values. No differences were seen in vehicle (DMSO solution) and URB597 injected eyes (**B**). Data was averaged as DMSO (dark grey) and URB597 injection (light grey) monkeys. Dashed light grey lines represent the baseline response at all frequencies for amplitudes and latencies (**A**,**B**). Normalized URB597 amplitude responses (post-URB597 amplitude minus post-DMSO amplitude) is shown following URB597 intravitreal injection (**C**). URB597 was significantly different than DMSO at 30 Hz. * *p* < 0.05; ** *p* < 0.01. Error bars represent standard error of the means (SEM).

**URB597 increases the amplitude of the photopic cone flicker ERG.** We have collected ERG recording pre-injection from all monkeys in order to validate that the ERG frequency-response curve was consistent with earlier studies in humans [49] (Figure 2). To study the effect of URB597 per se, the photopic flicker ERG was recorded in vehicle (DMSO solution) and URB597 (diluted in DMSO) intravitreal injected monkeys at four different frequencies, ranging from 15 Hz to 30 Hz, obtained in light-adapted conditions (30 cd.m^2^) with standard intensity light flashes (2.57 cd.s.m^2^). Figure 3A shows the frequency-response relationship of the flicker ERG before injection and following URB597 and DMSO injection. There was no significant difference in implicit time values between the two groups of intravitreally injected monkeys (Figure 3B). Before injection, recorded baseline amplitude responses were 192.68 ± 20.44 µV at 15 Hz, 172.59 ± 23.32 µV at 20 Hz, 154.05 ± 23.34 µV at 25 Hz and 126.98 ± 28.55 µV at 30 Hz. Following the injection of the vehicle (DMSO), recorded baseline amplitudes were 179.78 ± 14.75 µV at 15 Hz, 134.18 ± 18.38 µV at 20 Hz, 110.62 ± 16.90 µV at 25 Hz and 83.46 ± 3.72 µV at 30 Hz. In the case of URB597 (dissolved in DMSO) injected monkeys, recorded amplitude responses were 181.41 ± 24.51 µV at 15 Hz, 153.44 ± 19.78 µV at 20 Hz, 134.11 ± 15.60 µV at 25 Hz and 109.81 ± 15.99 µV at 30 Hz. Normality was checked with Q-Q Plots. No deviations were noted. Mauchly’s test was significant (χ^2^(5) = 11.769, *p* = 0.039), indicating that the assumption of sphericity was violated. Therefore, these responses were analyzed with a 2 (between subject factor; administered drug: DMSO versus URB597) × 2 (within subject factor; time of recording: pre-versus post-injection) × 4 (within subject factor; frequencies: 15, 20, 25 and 30 Hz) mixed factor ANOVA with Greenhouse–Geisser correction to the degrees of freedom (ε = 0.713). There were significant main effects of the time of recording (F(1, 10) = 9.264, *p* < 0.05 *, η^2^*p* = 0.481) and frequencies (F(2.442, 24.421) = 93.897, *p* < 0.001 ***, η^2^*p* = 0.904), but not of injected substance (F(1, 10) = 1.353, *p* = 0.272, η^2^*p* = 0.119) on overall amplitude responses. However, there was a significant two-way interaction between time of recording and frequencies, F(2.139, 21.389) = 4.991, *p* = 0.015, η^2^*p* = 0.333, as well as a significant three-way interaction between time of recording, frequencies and injected substance, F(2.139, 21.389) = 4.639, *p* = 0.019, η^2^*p* = 0.317. Simple main effects analysis revealed that while ERG responses did not differ between the two groups of monkeys before injection, ERG responses were significantly higher in URB597 injected monkeys than those in DMSO injected monkeys at the frequencies of 25 Hz (F(1) = 5.141, *p* = 0.047 *) and 30 Hz (F(1) = 10.897, *p* = 0.008 **). These findings revealed that, when compared to the injection of the DMSO vehicle, injection of URB597 increases ERG responses at 25 and 30 Hz (see Figure 3C), an effect that was most significant at 30 Hz, a frequency known to elicit only the response of the cone system.

## 4. Discussion

Our results showed higher photopic flicker ERG amplitudes following intravitreal injection of URB597 at 25 and 30 Hz. This corroborates our previous study using full-field ERG recordings and cannabinoid receptor blockers [14]. We do not consider that the IOP had an influence on the ERG components given that there was no statistical difference before and after intravitreal injections, especially after injection 50 µL of liquid. Higher ERG recordings might represent an increase in retinal sensitivity to light. This is in accordance with the fact that CB1R modulates synaptic release of glutamate in cone photoreceptors [7]. Activation of CB1R in any neuronal cell induces a decrease in synaptic release of neurotransmitters [50]. In our model of photopic flicker ERG testing in anesthetized monkeys, we could hypothesize that URB597 suppresses the release of glutamate by decreasing eCBs degradation, and therefore increasing sensitivity. It is important to remind here that photoreceptors respond to light by decreasing the release of neurotransmitters. Therefore, URB597 acts by increasing the threshold of response of phototransduction following light absorption.

It is well documented that the main physiological generator of the photopic 30 Hz flicker ERG is the cone system with post-receptoral On and Off pathways [18]. At this rate, rod photoreceptors are bleached and do not respond. Even under light adapted conditions, there is an initial onset from light saturated rods and are not included in our analysis. The general appearance of the ERG amplitude with rising flicker frequency function resembles that of the human eye, between 15 Hz and 30 Hz [49]. CB1R had an effect at 30 Hz frequency corresponding to the cone system suggesting that it is indeed the cone pathway that is involved in generating the amplitude responses. It is important to point out that the resulting increase in AEA levels due to the inhibition of FAAH by URB597 leads to the activation of CB1R, and therefore to an altered flicker ERG cone response. Activation of TRPV1 in the horizontal pathway of the vervet monkey retina, namely the horizontal and amacrine cells, by AEA can also play a role in the higher photopic flicker amplitudes observed here. However, it would be interesting to test if the horizontal pathway is activated using intraocular agonists/antagonists in contrast perception optometry tests.

The physiological effects of intravitreal URB597 in this study may be mediated via cannabinoid signaling in cones since eCBs, including AEA, can act at presynaptic CB1R to modify the activity of cone pedicles channels, resulting in a decrease in glutamate release. Consistent with this, using ffERG, although intravitreal administration of AM251 yielded statistically higher amplitude responses when averaged across flash intensities, it is important to note that around 0 log cd.s.m^−2^, we can see a tendency towards a decrease amplitude response [14]. Therefore, as URB597 increases levels of AEA, our results suggest the effects associated with this drug may be CB1R-mediated, resulting in part from reduction of glutamate via reduced neurotransmitter release. It has been shown that chronic use of the FAAH inhibitor results in a pronounced upregulation of AEA, which could also activate GPR55 [51]. However, we can rule out that our effects act through this receptor given that GPR55 is exclusively expressed in rods of vervet monkeys and, at our recording settings (at 30 Hz frequency), the rod system is saturated. Furthermore, we should underline that at a frequency of 15 Hz, both rods and cones are activated and, therefore, is possible that URB597 might influence the rod response. This is unlikely since at 15 Hz, we found no significant differences in ERG response amplitudes.

**Figure 4:**
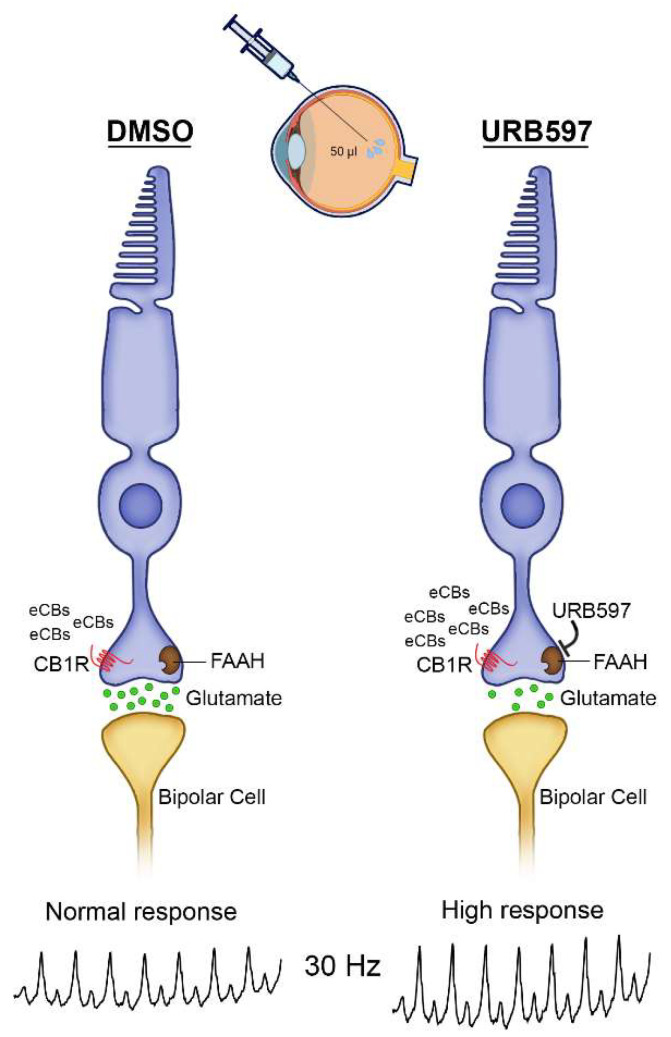
Overview of the putative mechanism of action of URB597 in the monkey retina. CB1R, cannabinoid receptor type 1; DMSO, dimethyl sulfoxide; eCBs, endocannabinoids; FAAH, fatty acid amide hydrolase (brown squares); glutamate (green filled circles); URB597.

**Hypothetical mechanism of action of URB597.** Our results suggest that the fatty acid amide hydroxylase in the monkey cone system regulates photopic vision. In light conditions, the rod system is saturated and the cone system is activated by different light frequencies in order to perceive our environment. There are anecdotal reports that found that color and photosensitivity are enhanced following cannabis consumption. Our data suggest that in these conditions, high concentrations of THC bond to CB1R as a true agonist, and thus can act similarly to light stimulation, and therefore perception. However, whether or not increases of amplitudes reflect necessary increases in vision perception remains unclear. Figure 4 illustrates a hypothetical mechanism of action of URB597 and their subsequent molecular effects in the vervet monkey retina. Our data support the idea that the eCB system plays an important role in retinal function. The amplitudes of the photopic flicker ERGs were higher when the FAAH was inhibited by URB597 in the injected eye but not in the vehicle DMSO-injected eyes shortly after an intravitreal injection. It might be interesting to investigate the long-term influence of cannabinoid agents in healthy, or diseased retina such as age-related macular degeneration, diabetic macular edema or retinitis pigmentosa.

**Methodological considerations.** Under light adapted conditions (30 cd/m^2^ for approximately 2 min), 30 Hz flicker ERGs selectively reflect cone activity, given that the rod system does not respond at this rate [18]. However, a typical frequency–response curve includes a mixed rod-cone response at the peak (15 Hz) vs. a pure cone response at 30 Hz. It would have been interesting to measure the effects of URB597 in pure scotopic conditions to rule out its implication in night vision. We found that our pre-injection baseline was higher than both DMSO or URB597 injections. DMSO by itself is known to have an acute effect on the ERG (0–60 min post-injection) [52], and further studies might be warranted to measure the time course of URB597 effects. Finally, by using URB597, we cannot exclude other actions played by fatty acid primary amides, N-acyltaurines, N-acylglycines, and 2-AG acting upon PPARalpha, GPR119, TRPV1, and CB2R [53].

## Figures and Tables

**Table 1:** Characteristics of the monkeys used in this study.

Animal Subjects				Drug
	ID	Sex	Weight	
**1**	o2011-3-7	female	3.33	Vehicle
**2**	o1097-1-3-8	female	3.23	Vehicle
**3**	o1787-4-5-4	female	3.48	Vehicle
**4**	o1085-7-2-1	female	3.36	Vehicle
**5**	o1842-6-5-3	male	4.28	Vehicle
**6**	o1986-2-0-5	male	3.29	Vehicle
**7**	o1085-7-7	female	3.03	URB597
**8**	o1313-1-2-2-3-2	female	2.87	URB597
**9**	o1669-3-7-1	female	2.95	URB597
**10**	o1645-1-7-2	female	2.7	URB597
**11**	o1083-13-8	female	3.06	URB597
**12**	o1842-4-2-1-2	male	3.1	URB597
**13**	o8711-8-4-1	male	2.8	URB597

## Data Availability

Data supporting reported results can be requested from J.B. (joseph.bouskila@umontreal.ca).
